# Ten-year indolent evolution of a Leydig cell tumor: A case report and literature review

**DOI:** 10.1016/j.eucr.2026.103414

**Published:** 2026-03-17

**Authors:** Charles Posite, Biruk Legesse, Mahad Said, Mirna Batista, Alexandre Amini, Bienfait Mumbere

**Affiliations:** a Department of Pathology, Kampala International University Western Campus, Bushenyi, Uganda; b Department of Pathology, Université Catholique du Graben, Butembo, Democratic Republic of the Congo; c Department of Urology, Université Catholique du Graben, Butembo, Democratic Republic of the Congo; d Department of Surgery, Kampala International University Western Campus, Bushenyi, Uganda

**Keywords:** Leydig cell tumor, Sex cord-stromal tumor, Testicular neoplasms

## Abstract

Leydig cell tumors (LCTs) are rare sex cord-stromal neoplasms that pose diagnostic challenges due to their overlap with germ cell tumors. This report describes a 45-year-old male with a 10-year history of a slow-growing, painless testicular mass. Preoperative imaging revealed a hypervascular, hypoechoic nodule. Histopathological analysis following a radical orchiectomy confirmed a benign LCT characterized by polygonal cells with eosinophilic cytoplasm and absent mitotic activity. The case emphasizes the shift toward early detection via ultrasound and highlights the necessity of long-term surveillance to monitor the metastatic potential inherent in adult LCTs.

## Highlights

• Reports a rare benign Leydig cell tumor in a 45-year-old asymptomatic male.
• Hypervascular, hypoechoic ultrasonography features aided early tumor detection.
• Histopathology confirmed pT1a staging with a distinct absence of mitotic activity.
• Discusses radical orchiectomy versus the trend toward testis-sparing surgery.
• Emphasizes the necessity of long-term surveillance for adult stromal neoplasms.

## 1 Introduction

Testicular neoplasms represent a sporadic group of tumors in men, with the rarest subset consisting of sex cord-stromal tumors.^1^ Among these, Leydig cell tumors (LCTs) are the most common entity.^2^ Historically, LCTs were considered exceptionally rare, accounting for approximately 1% to 3% of all testicular neoplasms.^3^ However, recent data suggest a rising incidence, with some studies reporting that LCTs may now comprise a significantly higher percentage (74.7%) of small testicular nodules.^4^ This apparent increase is largely attributed to the widespread clinical application of high-resolution ultrasound technology, which facilitates the early detection of small, asymptomatic lesions that might have remained undetected in historical series.^3^^,^^5^

LCTs are non-germ cell tumors that originate from the interstitial tissue of the testis. They exhibit a bimodal age distribution, typically manifesting in preadolescent children or in adults between the third and sixth decades of life.^6^ While the vast majority (approximately 90%) of these tumors are benign and localized to the testis, approximately 10% in adults exhibit malignant behavior with the potential for metastatic spread.^7^

The clinical presentation of LCTs is often characterized by a slow-growing, painless testicular mass; however, atypical presentations such as hydrocele have also been documented.^8^ Because Leydig cells are responsible for androgen production, these tumors can present with various endocrine manifestations. In prepubertal boys, this typically involves isosexual precocious puberty.^6^ In adults, patients may present with symptoms related to altered hormone levels, such as gynecomastia, decreased libido, or primary infertility, particularly when associated with risk factors like cryptorchidism.^1^

Diagnosing LCTs preoperatively remains a challenge, as they must be differentiated from more common germ cell tumors. Because percutaneous biopsy is generally avoided to prevent tumor seeding, clinicians rely on physical examination, serum tumor markers, and multimodal imaging.^5^ Ultrasound typically reveals a well-defined, hypoechoic, and hypervascular lesion.^9^ While radical inguinal orchiectomy remains the standard of care for suspected malignancy, the predominantly benign nature of small LCTs has led to the increased use of testis-sparing surgery (TSS) to preserve endocrine function and fertility.^4^^,^^9^ This case report details the management of a 45-year-old male with LCT, highlighting the diagnostic process and the importance of long-term surveillance.

## 2 Case presentation

In March 2025, a 45-year-old male presented to the Surgical Department of Kampala International University Teaching Hospital with a 10-year history of a slow-growing, painless mass in the left testis. The patient reported no associated constitutional symptoms, such as weight loss or fever, and denied any reduction in libido, history of trauma, and history of breasts enlargement, or prior scrotal infections. His personal and family medical histories were non-contributory, specifically regarding cryptorchidism or urogenital malignancies. Physical examination revealed a palpable, firm, non-tender nodule within the left testis; the contralateral testis and epididymis appeared normal. No gynecomastia or signs of endocrine dysfunction were clinically evident.

Serum testosterone was 900 ng/mL (264-916 ng/mL), estradiol 35 pg/mL (10-40 pg/mL), AFP 5 ng/mL (<10 ng/mL), β-hCG 0.5 mIU/mL (<1 mIU/mL), and LDH 175 U/L (100-250 U/L). Scrotal ultrasonography identified a well-circumscribed, homogeneous, hypoechoic intraparenchymal left testicular mass measuring 1.4 × 1.0 cm. Color Doppler imaging demonstrated significant internal hypervascularity. The diagnosis of a testicular tumor was made. Given the chronicity and localized nature of the lesion, the patient was counseled on surgical options and subsequently consented to a left radical orchiectomy.

**Fig. 1:**
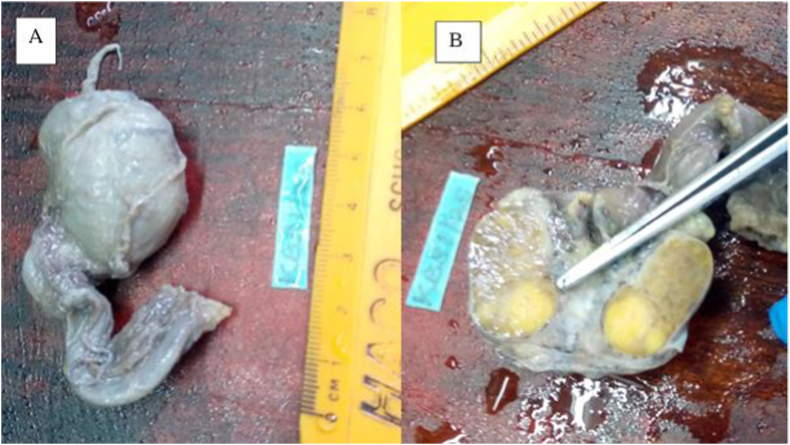
Testicular specimen measuring 2.7 × 1.6 × 1.3 cm (A). The cut surface shows a sharply demarcated, solid, yellowish nodule of 1.4 × 1.0 cm (B).

The surgical specimen consisted of a testis (2.7 × 1.6 × 1.3 cm), with an attached epididymis and a spermatic cord (3.5 cm). Sectioning of the testis revealed a demarcated, solid, yellowish nodule (1.4 × 1.0 cm). The lesion was devoid of hemorrhage or necrosis, and the remaining testicular parenchyma appeared grossly unremarkable (Fig. 1).

**Fig. 2:**
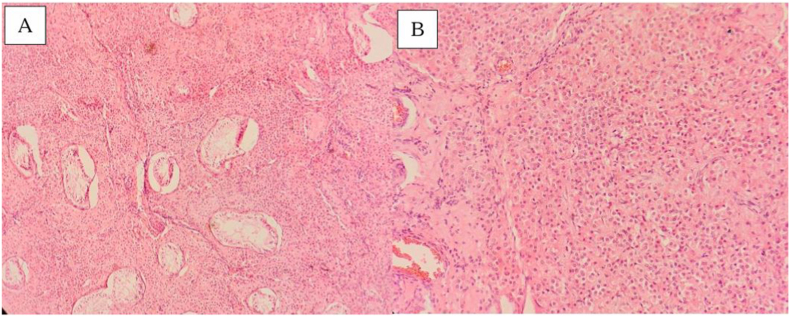
Hematoxylin and Eosin-stained sections show the diffuse architectural pattern of the tumor composed of large, polygonal cells with abundant, eosinophilic granular cytoplasm, uniform and round nuclei, and prominent central nucleoli (A-x100 & B-x200).

Histopathological analysis confirmed a unifocal sex cord-stromal tumor. The lesion exhibited a diffuse architectural pattern composed of large, polygonal cells characterized by abundant, eosinophilic granular cytoplasm. The nuclei were uniform and round, featuring prominent central nucleoli. While vascular proliferation and compression of the adjacent seminiferous tubules were observed, there was a distinct absence of nuclear atypia, mitotic activity, or necrosis (Fig. 2). The tumor remained limited to the testis. Reinke crystals were absent. The spermatic cord surgical margin was free of neoplastic cells, and no lymphovascular invasion was identified. Based on these findings, the lesion was diagnosed as a Leydig Cell Tumor, and it was staged as pT1a Nx. Due to limited local availability, immunohistochemical analysis was not performed. However, the diagnosis was established through histopathological features.

The postoperative recovery was uneventful, and the patient was discharged shortly after the procedure. Following the favorable histological profile of the LCT, a surveillance protocol was established. At the 12-month follow-up, which included semi-annual clinical and abdominal ultrasound assessments, the patient remained asymptomatic with no evidence of local recurrence or metastatic disease.

## 3 Discussion

LCTs are rare sex cord-stromal neoplasms that, despite their low overall incidence, represent a significant diagnostic and therapeutic consideration in the management of testicular masses.^10^ The current case involving a 45-year-old male highlights several characteristic features of LCTs while underscoring the complexities involved in differentiating benign from malignant phenotypes.

LCTs typically exhibit a bimodal distribution, peaking in children (5-10 years) and adults (20-60 years).^2^^,^^6^ While these tumors often present with endocrine disturbances (such as precocious puberty in children or gynecomastia and infertility in adults due to the aromatization of androgens to estrogens), our patient remained endocrinologically asymptomatic.^1^ The 10-year history of a slow-growing mass in this patient further reinforces the typically indolent nature of benign LCTs.

The preoperative diagnosis of LCT remains challenging because imaging characteristics often overlap with germ cell tumors (GCTs). Ultrasonographically, LCTs are usually well-circumscribed, hypoechoic, and hypervascular.^5^ In this case, the homogeneous hypoechoic nature and significant internal hypervascularity on Doppler imaging were consistent with established literature.^9^ However, as these features are not pathognomonic, radical orchiectomy is frequently performed when malignancy cannot be definitively excluded, as was the clinical decision in this instance.^9^^,^^10^

The definitive diagnosis of LCT rests on histopathological evaluation. Characteristic microscopic findings include large polygonal cells with eosinophilic granular cytoplasm and prominent nucleoli. Though degraded by formalin fixation, Reinke crystals, pathognomonic, may be identified in 30% of cases.^7^ Unlike GCTs, which are staged primarily by anatomical spread, the malignant potential of LCTs is predicted by specific histological criteria. Kim's criteria for malignancy include a tumor diameter >5 cm, infiltrative margins, necrosis, high mitotic activity (>3 mitoses per 10 HPF), vascular invasion, and significant nuclear atypia.^3^ Our patient's tumor was small (1.4 cm) and lacked mitotic activity or necrosis, suggesting a benign clinical course, which is categorized as pT1a.^7^

While radical inguinal orchiectomy was performed in this case, there is a growing consensus favoring TSS for small (<2 cm), polar, and ultrasound-benign appearing masses, provided intraoperative frozen section analysis is available.^4^^,^^9^ TSS offers the advantage of preserving hormonal function and fertility, which is particularly relevant in younger populations. However, the decision for radical surgery in our patient was supported by the necessity of ensuring complete oncological clearance in a setting where the “grey zone” of malignancy risk could not be entirely mitigated preoperatively, in addition to the lack of intraoperative frozen section analysis.^3^

Given that approximately 10% of adult LCTs may metastasize (often to retroperitoneal lymph nodes, the liver, or lungs), long-term surveillance is mandatory.^7^ Malignant LCTs are notoriously resistant to conventional chemotherapy and radiotherapy, making early detection of recurrence through serial imaging and clinical exams the primary focus of postoperative care.^8^ Our patient's stable condition at the 12-month follow-up is encouraging and consistent with the favorable prognosis associated with LCTs lacking high-risk histopathological features.^10^

## 4 Conclusion

Leydig cell tumors represent a rare but significant subset of testicular neoplasms that frequently present as indolent, hormonally silent masses. This case reinforces the diagnostic challenge of differentiating these lesions from germ cell tumors preoperatively, as current imaging modalities and serum markers often lack pathognomonic specificity. While histopathological evaluation remains the gold standard for risk stratification, the absence of high-risk features in this patient supports a benign prognosis. Furthermore, this case highlights the surgical paradigm shift toward testis-sparing surgery for small, localized lesions to preserve endocrine function and fertility, although radical orchiectomy remains a definitive measure for ensuring oncological safety in a clinical “grey zone”. Given the metastatic potential of approximately 10% of adult cases and their general resistance to adjuvant therapies, rigorous long-term surveillance is essential to ensure favorable outcomes and timely detection of recurrence.

## CRediT authorship contribution statement

**Charles Posite:** Writing – review & editing, Writing – original draft, Conceptualization. **Biruk Legesse:** Writing – review & editing, Supervision. **Mahad Said:** Writing – review & editing. **Mirna Batista:** Writing – review & editing, Supervision. **Alexandre Amini:** Writing – review & editing. **Bienfait Mumbere:** Writing – review & editing, Supervision.

## Patient consent

Written informed consent was obtained from the patient for publication of this case report and accompanying images. A copy of the written consent is available for review by the Editor-in-Chief of this journal on request.
